# UMD-MLH1/MSH2/MSH6 databases: description and analysis of genetic variations in French Lynch syndrome families

**DOI:** 10.1093/database/bat036

**Published:** 2013-05-31

**Authors:** Philippe Grandval, Aurélie J. Fabre, Pascaline Gaildrat, Stéphanie Baert-Desurmont, Marie-Pierre Buisine, Anthony Ferrari, Qing Wang, Christophe Béroud, Sylviane Olschwang

**Affiliations:** ^1^UMR_S910, INSERM, Marseille, France, ^2^AP-HM La Timone, Gastroenterology Department, Marseille, France, ^3^Cancer Biology Department, Institut Paoli-Calmettes, Marseille, France, ^4^Department of Genetics, University Hospital; Institute for Research and Innovation in Biomedicine, Rouen University; Inserm, U1079 Rouen, France, ^5^Biochemistry and Molecular Genetics Department, CHRU Claude Huriez, Lille, France, ^6^Centre Léon Bérard and Synergie Lyon Cancer, Lyon, France, ^7^Faculté de Médecine de la Timone, Université de La Méditerranée, Marseille, France and ^8^Department of Gastroenterology, Ambroise Paré Hospital, Marseille, France

## Abstract

Lynch syndrome is an autosomal dominant disease caused by germ line heterozygous mutations mainly involving the *MSH2*, *MLH1* and *MSH6* genes that belong to the DNA MisMatch Repair (MMR) genes family. The French network counting the 16 licensed laboratories involved in Lynch syndrome genetic testing developed three locus-specific databases with the UMD® software (www.umd.be/MLH1/, www.umd.be/MSH2/ and www.umd.be/MSH6/) that presently contain a total of 7047 sequence variations including 707 distinct variations of *a priori* unknown functional significance (VUS) that were identified through complete mutation screening or targeted predictive testing. Mutation carriers are at high risk for developing early-onset colorectal and endometrial adenocarcinomas. Consensus clinical guidelines have been proposed, allowing the efficient detection of curable lesions. The major challenge of genetic testing is to reliably classify the genomic variations in those patients who seek genetic counseling. Combining the interactive tools of the software, the relevant published data and mainly original information produced by the French MisMatch Repair network, the UMD-MLH1/MSH2/MSH6 databases provide interpretation data for the 707 VUS that were classified according to the IARC 5-Class system. These public databases are regularly updated to improve the classification of all registered VUS, exploring their role in cancer pre-disposition based on structural and functional approaches.

## Introduction

Mismatch repair (MMR) genes mutation carriers are a subgroup of colorectal cancer prone individuals who, when identified, benefit from highly effective risk management (1). The genetic condition, Lynch syndrome, shows autosomal dominant inheritance and incomplete penetrance. This condition is genetically heterogeneous, as at least four MMR genes (*MSH2*, *MLH1*, *MSH6* and *PMS2*) are implicated. To date, most of the causative mutations have been identified in two of them, *MSH2* and *MLH1*. It has been estimated that the prevalence of mutations in either of these two genes in Western countries is between 1 of 500 and 1 of 1000. Monitoring of microsatellite DNA instability (MSI) in tumor cells provides good sensitivity but much lower specificity for the diagnosis of the syndrome. This instability is caused by the bi-allelic inactivation of the *MLH1*, *MSH2*, *MSH6* or *PMS2* genes in patients who may or may not carry a germ line mutation in these genes.

Amsterdam criteria have been first established to identify such condition based only on patients’ personal and family history of colorectal cancer. These initial criteria were too stringent to detect Lynch patients in medical practice and were progressively enlarged to include the MSI status of an extended spectrum of tumor types (2). Indeed, although mutation carriers have been shown to be mainly at high risk of colorectal and endometrial adenocarcinomas, they are also exposed to an increased risk for cancer of small bowel, upper urological tract, stomach, ovary and biliary tract. Overall increased cancer risk is estimated to start at age 25–30 years. Cumulative cancer risk is ∼0.70 at 70 years (3, 4).

Unclear or misleading laboratory reports may have major clinical implication, as the presence or absence of a pathogenic MMR mutation typically impacts the patients’ management process. In the past 8 years, external quality controls emphasized the importance of an established knowledge of the genes being tested by the diagnostic personnel to guarantee the reliable conclusion of genetic testing (5). Mutations predicted to result in protein truncation, i.e. nonsense mutations, short deletion/insertion mutations lying within one exon associated with a frame shift, mutations involving nucleotides ±1 and ±2 within splicing junctions and large genomic rearrangements, are highly likely to impair the MMR function and are definitely classified as causing Lynch syndrome without additional information. As proposed by the Unclassified Genetic Variants Working Group (6), all other genomic variations are *a priori* classified of unknown functional significance (VUS) and require further investigation to document their impact on the MMR function. To refine the functional consequence of such variations, several criteria are generally used. *In silico* predictions first give an orientation toward a more likely neutral or causal effect. In theses studies, degree of conservation at the variation position among species and gene families, physico-chemical consequences on the predicted variant protein and splicing effect are investigated (7, 8). Second, published reports and public databases, such as functional tests and single nucleotide polymorphisms records, are important to consider. Lastly, additional biological tests may be attempted to reach an unambiguous conclusion. A combined approach is therefore recommended to determine the contribution of VUS to Lynch syndrome (9).

Mutation databases can help in the classification of VUS by providing a compiled source of a large amount of information. We present here the databases of all genetic variations encountered by the French MMR network, which is made of the 16 licensed laboratories in France involved in the molecular characterization of Lynch syndrome, i.e. the MMR genes germ line analyses. These databases were developed with the universal mutation database UMD® software (10). They have been endorsed by the French Cancer National Institute INCa. They are available online (UMD-MLH1/MSH2/MSH6: http://www.umd.be/MLH1/, http://www.umd.be/MSH2/ and http://www.umd.be/MSH6/). Two curators collect and compile information from all 16 laboratories. The UMD-MLH1/MSH2/MSH6 databases centralize all identified variations whether causal, neutral or VUS; these variations are linked together through a unique sample ID, allowing the simultaneous retrieval of all genotypes reported in the databases for a given sample, called co-occurrences. In June 2012, the UMD-MLH1/MSH2/MSH6 databases contained data from 2389 entries for *MLH1*, 2380 for *MSH2* and 1711 for *MSH6*. In this report, we first analyze variations observed in the French population. Then, we describe the 707 distinct *MLH1*, *MSH2* and *MSH6* VUS and the associated clinical and biological data. Finally, we explore the co-occurrence data in an attempt to classify VUS as either causal or neutral.

## Methods

### MLH1/MSH2/MSH6-UMD databases

Supported by the French national cancer institute, the Universal Mutation Database core database was retained to register the MMR germ line variations observed in the French population (http://www.umd.be/, UMD: PMID10612827 and PMID16086365) (10, 11). The UMD generic software includes an optimized structure to assist and secure data entry and to allow the input of various clinical or biological data. Offline copies of UMD-MLH1/MSH2/MSH6 are continuously edited and updated by the curator; edited copies are regularly deposited on the server. These public databases are available online. Their integrity is ensured by the original template offline. The curators would gratefully receive notification of errors in the current version. Databases design complies with the general recommendations for Locus-Specific Data Bases (LSDB) characteristics and follow-up as proposed by the Human Genome Variation Society (12).

Each website is divided in six parts. The ‘gene’, ‘protein’ and ‘clinics’ are dedicated to information on the *MLH1*, MSH2 or *MSH6* genes (numbering of exons, cDNA sequences and information on the protein) and the Lynch syndrome. The ‘mutations’ and ‘statistics’ options allow the user to search into the databases. Hyperlinks are provided to access the data search options. The sixth part, ‘references’, aims at providing a list of all published references related to the VUS reported in the databases. It is currently in the validation process. The web pages also contain links to external information including other UMD databases and public central databases (Pubmed, OMIM, Unigene, GeneCard as examples).

The UMD knowledgebase system includes many analytical tools (10, 11). The user is able to optimize multicriteria search tools to select records from any field. Data can be displayed by several searching tools under the ‘mutations’ section such as the type of mutations, exon/intron location and biological category. Alternatively, the option ‘I found a mutation’ provides access to a quick search (nucleotide or amino acid position), and ‘free search’ provides access to an advanced search and a customized search interface with several items (sample ID, amino acid position, biological significance, etc.). A click on hyperlinks provides access to different lists of variations but not to downloading.

### Data collection

According to the French law #78-17, both project and on-line publishing were approved by the French supervisory authority CNIL (Commission Nationale pour l’Informatique et les Libertés, registration no. 908361, 17 July 2009) and the national ethics committee CCTIRS (Comité Consultatif pour le Traitement de l’Information en matière de Recherche dans le domaine de la Santé, approval no. 07.421, 22 November 2007). About 20 000 index cases were addressed to the French medical genetics departments between January 1995 and June 2012 for investigation of possible Lynch syndrome. Genetic testing was performed according to the eligibility criteria stated by the French consensus (13). All patients and at-risk relatives signed a written informed consent during a personal interview with a genetic counselor before genetic testing. Patients who were detected as mutation carriers received a specific information relative to the registration of their genetic results in the national database during a second interview.

The full coding sequences and exon–intron junctions of the *MLH1*, *MSH2* and *MSH6* genes were screened for variations, based on prescreening methods or more recently by systematic exon sequencing. Large genomic rearrangements were identified by Multiplex Ligation-dependent Probe Amplification or Quantitative Multiplex PCR of Short Fluorescent Fragments. The corresponding references are displayed in the ‘clinics’ section. The French MMR network of laboratories involved in the genetic testing of Lynch syndrome collected all MMR variations identified in blood samples of the affected patients and at-risk relatives.

Quality control procedures were in place throughout the collection, processing and storing of data as required by the standard ISO 15189 in all laboratories. Genotypes were double checked on two independent biological samples from the same individuals except in case of index case death before availability of genetic result. Reference sequences for the *MLH1*, *MSH2* and *MSH6* genes were NM_000249.3, NM_000251.1 and NM_000179.2, respectively, as mentioned in the Locus Reference Genomic database. Genomic variations were described according to recommendations of the human genome variation society (www.hgvs.org/mutnomen/), and the predicted proteins were deduced using the algorithms of the UMD core system. Data are freely accessible at www.umd.be/MLH1/, www.umd.be/MSH2/ and www.umd.be/MSH6/. An anonymous and unique sample identifier (sample_ID) was generated for each individual and was used in the three databases. In June 2012 release, 2562 entries were recorded for *MLH1*, 2731 for *MSH2* and 1754 for *MSH6*. The UMD software automatically checked for the correct description of the sequence variations at the nucleotide level and generated the variation name at the protein level. Collection of phenotypic data is in progress as well as pictures of Sanger-sequencing profiles. As the treatment of genomic events like large genomic deletions, insertions or deletions surrounding splicing junctions, deletion/insertions and variations located in the 5′UTR and 3′UTR regions, was recently integrated to UMD database structure, these variations are currently in the validation process and will be soon uploaded.

### Variations analysis

The system of five variation groups developed at the International Anticancer Research Center was used for functional classification (6). Mutations predicted to result in protein truncation were classified as deleterious and no longer discussed. The remaining variations were classified of VUS and were selected for additional investigation. Reports about these VUS either in publications or in other *MLH1/MSH2/MSH6* databases are regularly reviewed. Information is individually available by clicking on ‘I found a mutation’ of the ‘mutations’ section. It will be soon fully accessible in the ‘references’ section.

For each VUS, *in silico* prediction, including splicing analyses, conservation level, SIFT and UMD-Predictor estimates were directly integrated into the UMD database structure. They are automatically activated when opening the summary of the corresponding variation.

In addition, five pieces of information were asked to French contributors then evaluated according to their influence on MMR function impairment (14):

- Cosegregation analyses were performed in the relevant families whenever possible, and the cumulative LOD scores were calculated, but the corresponding pedigrees were not displayed to keep data anonymous;
- Clinical phenotype was detailed for each patient according to the enlarged Amsterdam criteria;
- Assessment of the MMR function in tumor cells by genotyping and immunohistochemistry analysis. MSI/MicroSatellite Stability (MSS) classification followed the consensus recommendations and was validated only if the proportion of cancer cells in the tumor fragment exceeded 10%. Immunohistochemistry was performed using antibodies against the two proteins MSH2 and MLH1, completed with MSH6 and PMS2 when possible;
- Splicing impairment was explored using a functional splicing assay developed and validated by Gaildrat *et al.* (15). In case of major abnormal splicing, confirmation by RT-PCR after mRNA extraction (PAXgene^TM^ Blood RNA Tube, Qiagen France) was required;
- Transcription-translation assays were performed for VUS involving the Kozak sequence or the 5′UTR.

Functional protein assays were traced through a systematic PubMed search that allowed collecting data from *in vitro* or *in/ex vivo* assays according to the classification proposed by Ou *et al.* (16). Allele frequencies were derived from on-line databases or specific studies when at least 100 control individuals (200 alleles) were tested.

All these data are displayed in a table entitled ‘Complementary data about this mutation’ following the links ‘mutations’, then ‘I found a mutation’ and then indicating the amino acid position.

Finally, the search for the presence of other variations found simultaneously within the three databases in the same sample_ID is currently implemented, and a co-occurrences table is automatically carried out on the same page. All data are also available following the links directly from the general table of mutations.

## Results

### UMD-MLH1/MSH2/MSH6 databases

In June 2012, the 16 licensed laboratories belonging to the French MMR network reported 2562 variations for *MLH1*, 2731 for *MSH2* and 1754 for *MSH6*. The data on frequent polymorphisms were not systematically collected. With the exception of large genomic deletions, insertions or deletions surrounding splicing junctions, deletion/insertions and variations located in the 5′UTR and 3′UTR regions that are not yet uploaded, 2389, 2380 and 1711 records could be integrated in the UMD-MLH1/MSH2/MSH6 databases, respectively. They represent 429 different variations on *MLH1*, 455 on *MSH2* and 290 on *MSH6*. These different variations were predicted to result in protein truncation (nonsense mutations, insertions or deletions associated with a frame shift, mutations involving nucleotides ±1 and ±2 within splicing junctions) in 171 (40%) occasions on *MLH1*, 199 (44%) on *MSH2* and 97 (33%) on *MSH6*. A total of 707 variations thus remained VUS.

**Figure 1.**
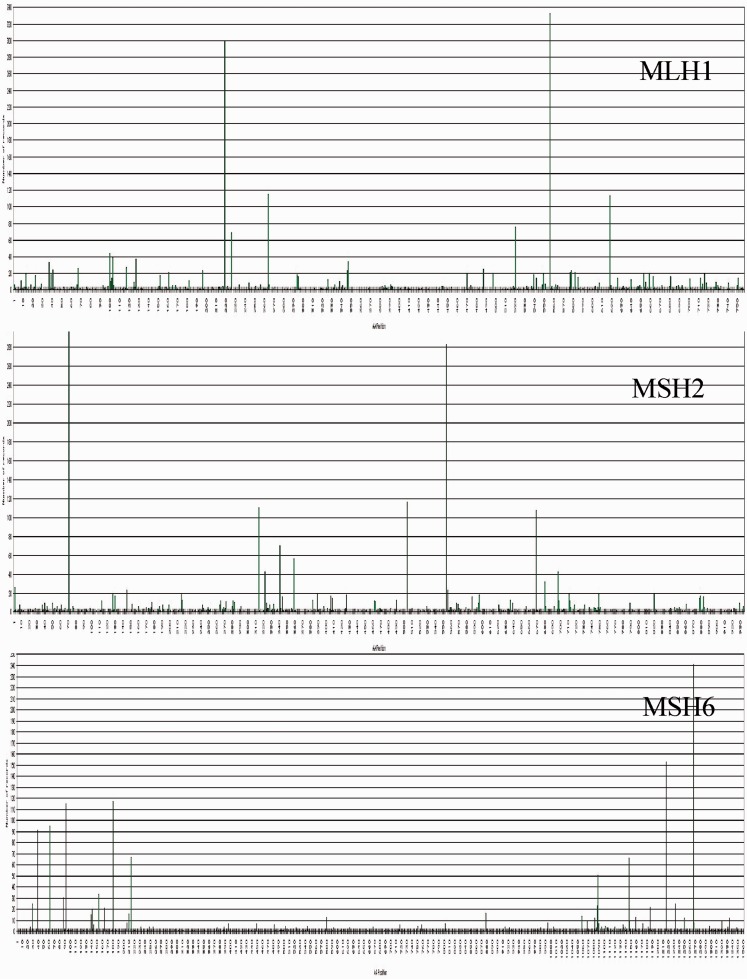
Graphical distribution of mutations recorded in the UMD-MLH1/MSH2/MSH6 databases. For each three genes, *MLH1*, *MSH2* and *MSH6*, from top to bottom, are symbolized the protein and its size (count of aminoacids) from the NH2- (left) to the COOH-end, then the cDNA from the 5′- (left) to the 3′-end and its size (count of nucleotides) and the histogram of presently recorded variations according to their position along the coding sequence. Vertical bars indicate the cumulative number of events at each position. These graphics and selections by exon or region are available following the links ‘statistics’ then ‘mutation map’. Other graphics are displayed when searching for frequencies or distributions.

Corresponding data can be sorted out as tables or individually viewed in the ‘mutations’ section. A graphical view of the mutations distribution along the coding sequence can be displayed in the ‘statistics’ section (Figure 1).

### Characterization of VUS

**Figure 2.**
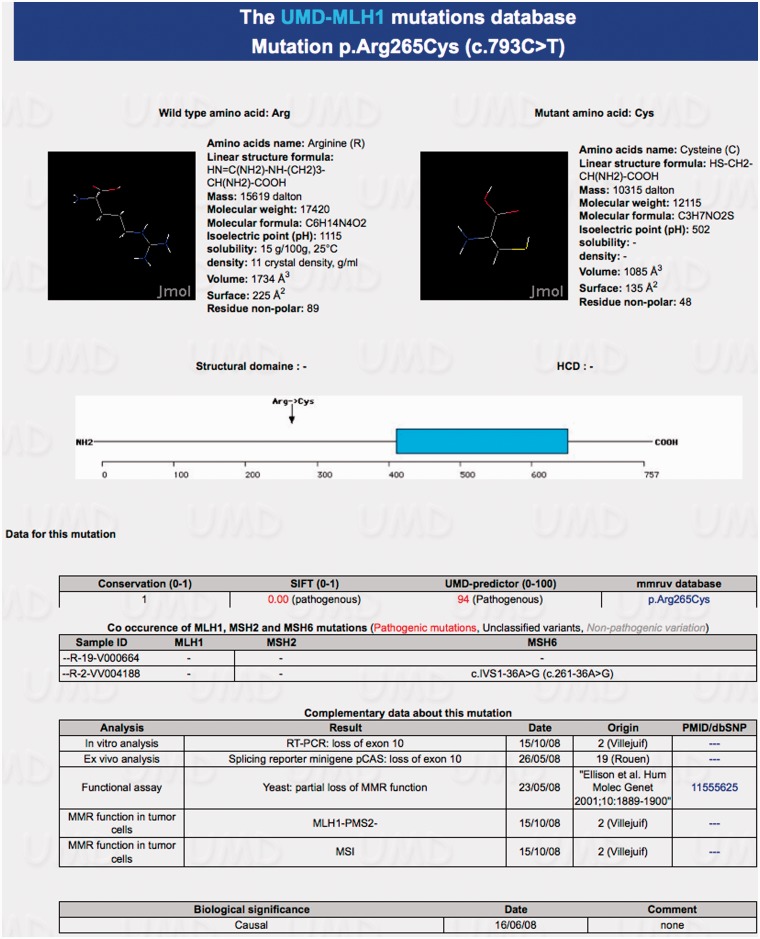
Information on the missense variation c.793C>T (p.Arg265Cys) of the *MLH1* gene. The physico-chemical properties of the normal and mutant aminoacids are first displayed with the position along the sequence. Data for this mutation are then given first regarding the *in silico* predictions and those data recorded in the mmruv database (by clicking on the aminoacid variation), then searching in the UMD-MLH1/MSH2/MSH6 databases for co-occurrences. Original data provided by the French MMR network are listed in a table together with the conclusion of published specific functional assays. Finally appears the consensus on the French MMR network on the biological significance of the variation regarding Lynch syndrome.

**Figure 3.**
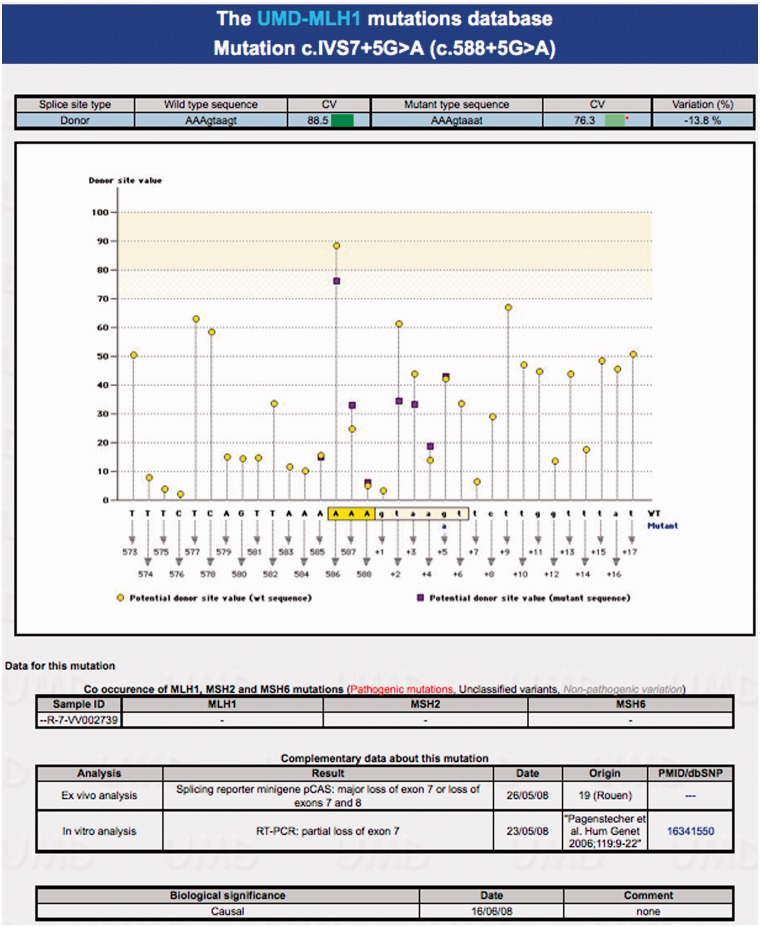
*In silico* analysis of the intronic variation c.588 + 5G>A surrounding the donor splicing junction of exon 7 of the *MLH1* gene. Normal and mutant genomic sequences are computed for their respective role in the splicing process. Consensus values are given in absence of mutation together with variations in yellow and purple, respectively, for nucleotide positions close to the mutation tagged in blue (here a g>a at position +5 of the intron). Nucleotides involved in the donor-splicing site are squared. Then follow the data available for this mutation as described in Figure 2.

The 707 VUS were scattered in 258 on the *MLH1* gene, 256 on *MSH2* and 193 on *MSH6*. In either the ‘mutations’ or ‘statistics’ option, *in silico* estimates can be displayed when clicking on a variation/variation type (missense, splicing sites, etc.) in a table or when asking for a specific position in the coding sequence or when searching using the different modules. When the non-truncating variation occurs within the coding sequence, the UMD-Predictor tool gives first an estimate of the functional impairment based on structural alteration of the corresponding protein, pathogenicity at the physic-chemical level together with the conservation level, the SIFT prediction and a graphical view of the amino acid involved in the variation (Figure 2). When the variation occurs within non-coding sequences located at the close vicinity of the splicing junctions (<10 nucleotides) or mid-intronic, prediction of the modification of the splicing process is graphically presented and summarized in a table giving Consensus Values and variation (Figure 3).

The co-occurrence data are extracted from the three databases simultaneously based on the sample ID. They consist in a table presenting all samples carrying the variation and a list of all variations entered for each sample in the three databases, using color codes to highlight deleterious variations (Figures 2 and 3).

Then the complementary data from the corresponding patients and tumors obtained by the French MMR network are listed regarding the clinical phenotype, the cosegregation within families, the status of MMR function in tumor cells and/or the splicing/protein characterization. These are original and mostly unpublished data. Each corresponding provider (i.e*.* the French contributing laboratory) is mentioned with the validation date of information. When available, data published by other groups are also displayed (Figures 2 and 3). A link toward the corresponding PubMed access has been incorporated to the relevant entry.

Finally, the consensus conclusion of the French MMR network regarding the role of the VUS in Lynch syndrome is stated as a functional class (neutral or class-1, likely neutral or class-2, causal or class-5, likely causal or class-4, VUS or class-3). It is reviewed when new data become available and only the most recent conclusion is presented and time stamped (Figures 2 and 3). The review of all 707 VUS reported in the UMD-MLH1/MSH2/MSH6 database allowed the French MMR network to classify 370 of them as causal (55 variations), likely causal (54 variations), likely neutral (80 variations) and neutral (181 variations) (Table 1).

**Table 1.** Distribution of the 707 MMR UVs according to their functional consequence on Lynch syndrome

Gene	*MLH1*	*MSH2*	*MSH6*
Class-5^a^ (55)	38	17	0
Class-4 (54)	27	17	10
Class-3 (337)	112	131	94
Class-2 (80)	16	33	31
Class-1 (181)	65	58	58

^a^Classes are defined according to Plon *et al.* (6). Class-1 corresponds to neutral variations, class-2 to likely neutral, class-3 to VUS, class-4 to likely causal and class-5 to causal.

## Discussion

### The UMD-MLH1/MSH2/MSH6 databases provide an overview of MMR variations present in the French population

We examined *MLH1/MSH2/MSH6* variations collected by the 16 licensed laboratories located in France and belonging to the French MMR network during the past 18 years. Updates on new variations or new samples found to carry MMR variations are done twice a year. In June 2012, a total of 7047 variations were provided for registration, and 6480 could be integrated by the UMD software for further analyses. They represent 1174 different variations corresponding to 467 truncating mutations (40%) and 707 VUS (60%). Entries correspond to all variations found through a complete exon screening of the three genes not only in index cases but also in those relatives that were genetically screened and found to be mutation carriers. This nation-wide and systematic registration should improve the prevalence estimate of germline MMR mutations in France. We do not know yet to what extent the index cases carrying the same mutation are genetically related, as haplotypes carrying the variant allele have not been constructed. We plan to progressively get haplotypes for all index cases from the French providers, an information that could help in obtaining reliable demographic parameters, in estimating mutation rates and in improving allele-specific lodscores.

### VUS classified by the French MMR network

A total of 337 of 707 non-truncating French variations remain VUS (48%) even with information reported in other databases and published works (see section ‘Variations analysis’). Additional experiments for all variations with uncertain functional status are requested from contributors. The most significant findings are used to change the functional status of the corresponding VUS according to the classification method previously proposed (14).

### Position of the UMD-MLH1/MSH2/MSH6 databases as public databases

Several MMR mutation databases already exist. The historical InSiGHT (International Society for Gastrointestinal Hereditary Tumors) database gives an exhaustive description of the data submitted by the contributors (www.insight-group.org). A dedicated database (www.mmruv.info) is linked to the previous one and contains the great majority of data available in the literature, but does not provide its own interpretation (16). The link from the UMD-MLH1/MSH2/MSH6 databases toward mmruv for all shared VUS is available by clicking on the amino acid change in the paragraph ‘data for this mutation’ (Figure 2). Another substantial work proposes a VUS classification based on a series of 932 early onset colorectal cancer patients registered for clinical data, MMR function in tumor cells, *in silico* analysis and genotyping studies in both relevant families and control population (17). A classification score is proposed, but the threshold indicating a pathogenic effect to be taken into account for patient management is not defined. Although the methodology for the score calculation is not given, we anticipate that this computation requires fulfilling all criteria, such situation being infrequent in routine practice. Finally, a bioinformatic prediction method called PON-MMR has been recently published, which proposes an online tool returning pathogenicity scores by email after submission of MMR missense variations (8). This tool is not connected to mutation databases or to related published data, thus precluding an easy review of eventual discordances.

## Conclusion

The UMD-MLH1/MSH2/MSH6 databases presented here are an integrated system, allowing the simultaneous access to selected published data and original information provided by the French MMR network in the three *MLH1*, *MSH2* and *MSH6* genes. Newly recorded information becomes available for consultation after consensus validation by the French working group. The databases are updated twice a year. In addition to the exhaustive registration of mutations found in the French population, the database presently contains the relevant data, allowing the classification of 370 variations either in (likely) neutral or causal. This classification has been achieved based on original data collection from patients referred to the French MMR network. The tool also displays all co-occurrences within the three genes on each VUS-specific page of the UMD-MLH1/MSH2/MSH6 databases. Information contained in the ‘mmruv’ database is available through a specific link on the same page. Pending data are progressively added, and attempt to classify the remaining 337 VUS is pursued. Therefore, the UMD-MLH1/MSH2/MSH6 databases consist in the most complete and comprehensive public tool for geneticists and molecular biologists to interpret their own sequencing results.
